# A Review on the Prevalence of *Toxoplasma gondii* in Humans and Animals Reported in Malaysia from 2008–2018

**DOI:** 10.3390/ijerph17134809

**Published:** 2020-07-03

**Authors:** Mohammed Nasiru Wana, Mohamad Aris Mohd Moklas, Malaika Watanabe, Norshariza Nordin, Ngah Zasmy Unyah, Sharif Alhassan Abdullahi, Ashraf Ahmad Issa Alapid, Tijjani Mustapha, Rusliza Basir, Roslaini Abd. Majid

**Affiliations:** 1 Department of Medical Microbiology and Parasitology, Faculty of Medicine and Health Sciences, Universiti Putra Malaysia, Serdang 43400, Selangor, Malaysia; mwnasiru@atbu.edu.ng (M.N.W.); ngah@upm.edu.my (N.Z.U.); sharifosis@gmail.com (S.A.A.); asalapid82@gmail.com (A.A.I.A.); tijjanimustapha@yahoo.com (T.M.); 2 Department of Biological Sciences, Faculty of Science, Abubakar Tafawa Balewa University Bauchi, 740272 Bauchi, Nigeria; 3 Department of Human Anatomy, Faculty of Medicine and Health Sciences, Universiti Putra Malaysia, Serdang 43400, Selangor, Malaysia; rusliza@upm.edu.my; 4 Department of Companion Animal Medicine & Surgery, Faculty of Veterinary Medicine, Universiti Putra Malaysia, Serdang 43400, Selangor, Malaysia; maraika@upm.edu.my; 5 Department of Biomedical Sciences, Faculty of Medicine and Health Sciences, Universiti Putra Malaysia, Serdang 43400, Selangor, Malaysia; shariza@upm.edu.my; 6 Department of Medical Microbiology and Parasitology, Faculty of Clinical Sciences, Bayero University Kano, 700241 Kano, Nigeria; 7 Department of Zoology, Faculty of Science-Alasaba, University of Gharyan, 010101 Gharyan, Libya; 8 Department of Biological Sciences, Faculty of Science, Yobe State University Damaturu, 620101 Damaturu, Nigeria; 9 Faculty of Medicine and Health, National Defence University of Malaysia, Kem Sungai Besi, Kuala Lumpur 57000, Selangor, Malaysia

**Keywords:** Malaysia, *Toxoplasma gondii*, toxoplasmosis, HIV patient, pregnant women, genotype, vaccine, prevalence, humans, animals

## Abstract

Toxoplasmosis is a disease caused by the protozoan parasite *Toxoplasma gondii* (*T. gondii*). Human toxoplasmosis seroprevalence in Malaysia has increased since it was first reported in 1973 as shown in previous reviews of 1991 and 2007. However, over a decade since the last review, comprehensive data on toxoplasmosis in Malaysia is lacking. This work aimed at reviewing articles on toxoplasmosis research in Malaysia in order to identify the research gaps, create public awareness, and efforts made so far and proffer management options on the disease. The present review examines the available published research articles from 2008 to 2018 related to toxoplasmosis research conducted in Malaysia. The articles reviewed were retrieved from nine credible databases such as Web of Science, Google Scholar, ScienceDirect, PubMed, Scopus, Springer, Wiley online library, Ovid, and Cochrane using the keywords; Malaysia, toxoplasmosis, *Toxoplasma gondii*, toxoplasma encephalitis, seroprevalence, human immunodeficiency virus (HIV) patients, pregnant women, genotype strain, anti-toxoplasma antibodies, felines, and vaccine. The data highlighted seropositive cases from healthy community members in Pangkor Island (59.7%) and among migrant workers (57.4%) at alarming rates, as well as 42.5% in pregnant women. Data on animal seroprevalence were limited and there was no information on cats as the definitive host. Genetic characterization of *Toxoplasma gondii* from HIV patients; pregnant women, and domestic cats is lacking. This present review on toxoplasmosis is beneficial to researchers, health workers, animal health professionals, and policymakers. Therefore, attention is required to educate and enlighten health workers and the general public about the risk factors associated with *T. gondii* infection in Malaysia.

## 1. Introduction

*Toxoplasma gondii* (*T. gondii*) is the cause of toxoplasmosis, which is a highly neglected disease that can be life-threatening in both humans and animals worldwide [1,2]. The disease can affect all mammals and birds, and the latter also play a significant role as a cause of zoonotic infection [3]. More than one-third of the population of the world is infected by *T. gondii* [4]. Primary infection is usually subclinical, although in immunocompromised patients, it may be life-threatening [5]. In healthy immunocompetent individuals, the infection is generally asymptomatic, but an atypical strain can be fatal [6], while in pregnant women it may lead to abortion [7]. Members of the family Felidae, which includes both wild and domestic cats, are the definitive hosts, while all other animals serve as the intermediate host of *T. gondii* [8,9]. There are several ways in which toxoplasmosis can be acquired, and these may include the ingestion of the oocysts infective stage, which is only shed by cats [10]. The oocysts in the outside environment may contaminate soil, water, and vegetables presenting a large source of infection [10,11,12,13]. Other routes of transmission include consumption of tissue cysts in undercooked meat of an infected animal, congenital via the placenta, from organ transplant, and through blood transfusion [14,15,16].

**Figure 1:**
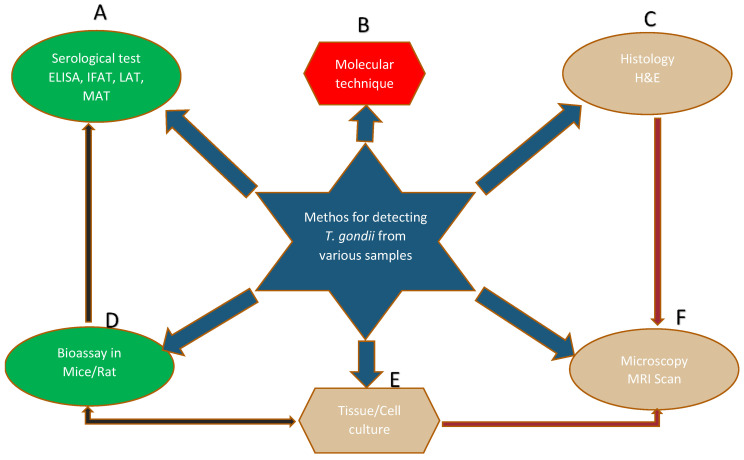
The letters A—F represent different methods for the diagnosis/detection of *T. gondii* in various samples. ELISA; enzyme-linked immunosorbent assay, IFAT; indirect fluorescence antibody test, LAT; latex agglutination test, MAT; modified agglutination test, H&E; hematoxylin and eosin, MRI; magnetic resonance imaging.

The diagnostic test for the detection of *T. gondii* (oocyst, tachyzoite, and bradyzoite) in a suspected tissues of humans or animals has evolved greatly over time, but serological test is more frequently common [17], followed by molecular techniques [18], and then histological techniques [19]. Other diagnostic methods with less reportage include bioassay in mice or cat, tissue culture, and microscopy [17]. Similarly, an innovative diagnostic approach and vaccine development against *T. gondii* has received attention in Malaysia [20]. A schematic diagram for the detection of *T. gondii* from various samples is shown in Figure 1.

**Figure 2:**
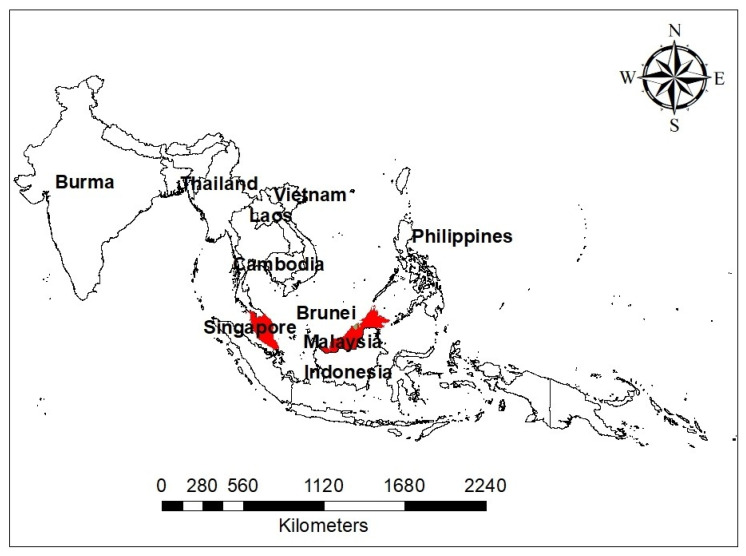
A map of Southeast Asia showing Malaysia colored red and its link with neighboring countries.

**Figure 3:**
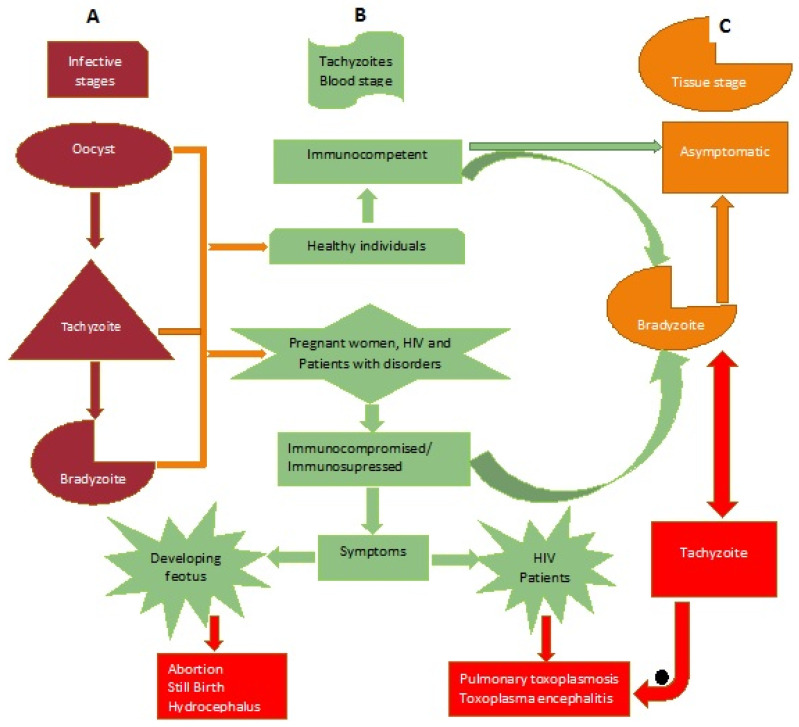
Distribution of *Toxoplasma gondii* and its effect on the human population. Column A: This represents the three infective stages. Column B: Group of individuals infected and the possible disease outcome. Column C: The chronic stage/latent toxoplasmosis with tissue cysts formation.

Malaysia is a small country located on the Southeast of Asia (Figure 2) that has an estimated population of 32.6 million people (https://www.dosm.gov.my/v1/index.php?r=column/cthemeByCat&cat=155&bul_id=aWJZRkJ4UEdKcUZpT2tVT090Snpydz09&menu_id=L0pheU43NWJwRWVSZklWdzQ4TlhUUT09). The country has three major ethnic groups comprising Malays, Chinese, and Indians. Malays are the dominant group, constituting more than 60% of the total population, and are known to keep domestic cats (a definitive host) as a pet, while most of the Indians and Chinese keeps dogs as pets [21]. The proposed pattern of *T. gondii* infection and its effect in both immunocompetent and immunocompromised individuals is presented in Figure 3. The development of pulmonary toxoplasmosis and/or toxoplasma encephalitis in immunosuppressed individual had been reported [22], while in immunocompetent individuals, pulmonary toxoplasmosis depends on the *T. gondii* strain causing the infection [6].

Nicolle and Manceaux in 1908 were the first to discover *T. gondii* in the spleen and bone marrow of rodent (*Ctenodactylus gundi*). Later on, research was conducted worldwide to decipher the mode of transmission, life cycle, method of diagnosis, prevention, and control. In 1973, the first case of human toxoplasmosis was reported in Malaysia as reviewed by Yahaya [23]. The review demonstrated an increase of prevalence from 13.9% to 30.2% within a period of 11 years. Further, the Malay ethnic group had higher levels of *T. gondii* infection compared to the Chinese and Indian ethnic groups. In addition, a review on human toxoplasmosis in Malaysia among different ethnic groups by Nissapatorn and Abdullah [21] showed an overall increase in prevalence to 45.8% in women with stillbirths and 51.2% in acquired immune deficiency syndrome (AIDS) patients. This similar trend of increased seroprevalence of human toxoplasmosis was also highlighted in the last review on ‘’Toxoplasmosis: A silent threat in Southeast Asia’’ by Nissapatorn [24]. Moreover, the recent review [25] of wild meat being traded in Malaysia has also documented *T. gondii* risk associated with wild animal meat consumption. Despite all the risks mentioned, toxoplasmosis research in Malaysia received little attention, even though there are large number of domestic cats (definitive host). The continuous rise in toxoplasmosis prevalence rate coupled with an increase in human immunodeficiency virus (HIV)/AIDS cases [26], the rise in population of migrant workers and complications during pregnancy should draw attention and consider *T. gondii* as a serious pathogen. Thus, it has become pertinent to review research in Malaysia related to *T. gondii* and toxoplasmosis in order to shed more light on the impact of the disease. Notwithstanding, effort to eliminate or eradicate the infection is still lacking and more often funding toward research is often limited. Data were sourced from nine credible electronic databases which include Web of Science, Google Scholar, ScienceDirect, PubMed, Scopus, Springer, Wiley Online library, Ovid and Cochrane. The keywords for the search were ‘Malaysia’ ‘Toxoplasmosis’, ‘*Toxoplasma gondii*’, ‘Toxoplasma encephalitis’, ‘Seroprevalence’, ‘HIV patients’, ‘Pregnant women’, ‘Genotype strain’, ‘Anti-toxoplasma antibodies’, ‘Felines’ and ‘Vaccine’. The present review attempts to provide information on the prevalence and risk factors of human and animal toxoplasmosis, progress made so far in vaccine development, and genetic characterization of *T. gondii* strains circulating in Malaysia to draw attention to this neglected disease. Further, the review provides direction and suggestions for future studies, prevention, and control of this life-threatening parasitic disease.

## 2. Methods for the Detection of *T. gondii* in Different Samples

The serological test measures the antibodies and determines the seroprevalence of infection by looking at the immunoglobulin G (IgG), immunoglobulin M (IgM), and IgG avidity levels in a sample, usually serum from the blood of specific host population. This is the simplest and easiest test but mostly is characterized by either false-positive or false-negative results [27]. The molecular techniques are robust, sensitive, and accurate tests [28]. They utilize all types of samples for the detection of a particular gene of interest unique to this particular organism. Various methods such as conventional polymerase chain reaction (PCR), quantitative-PCR (qPCR), and loop-mediated amplification (LMAP) are some of the methods frequently employed [29]. Histological techniques are the method rarely used. It focuses mostly on detecting the bradyzoite stage in tissues such as the heart, liver, and brain [30]. Such tissues will be fixed onto the glass slide and stained with hematoxylin and eosin (H&E) before viewing through a microscope. Bioassay/in vivo using an animal model (mice/rat) is another way of testing suspected samples such as feline feces, liver, lung, and brain homogenate of intermediate host through inoculation and subsequently test the animal for the presence of an infection [31]. The test is expensive and time consuming but is a reliable way to measure the viability and virulence factor of the different strains. The in vitro/tissue culture approach eliminates the use of animals through the provision of an artificial environment where suspected samples such as blood are grown in a media [32]. The endpoint of tissue culture requires microscopy to measure the motility or viability of the sample. Microscopy is still the cornerstone of most intuitive discoveries that can detect the morphology of the parasite. Its versatility is always relied upon by other tests such as tissue culture and histology [33].

## 3. Comparison of Serological Methods for the Detection of *T. gondii* Antibodies in Malaysia

Convenient sampling was used for data collection in all studies and two serological tests of enzyme-linked immunosorbent assay (ELISA) and indirect fluorescent antibody test (IFAT) were used to evaluate results based on the detection of IgG, IgM, and avidity test of *T. gondii* antibodies (Table 1). The protocols for specimen collection and processing were mostly not standardized in all studies, while in the majority, information on the control group was not available. However, all the ELISA and IFAT kits used were manufactured from different companies making it difficult to compare and validate their specificities and sensitivities [34]. Further confirmation of results through PCR was not done except in one study. Therefore, some of the results were doubtful due to discrepancies in experimental design and the commercial kits used.

## 4. Human Seroprevalence of Toxoplasmosis in Malaysia 

Research has been documented in Malaysia and other neighboring countries that evaluated the seroprevalence of *T. gondii* among different individual groups. The majority of the studies have focused on the seroprevalence in pregnant women [35,36], followed by patients with disorders [37,38], healthy community members [39,40], migrant workers [41,42], and schizophrenic patients [43,44]. In addition, these studies also focused on the socio-demographic data, epidemiological profile, possible risk factors of acquiring the *T. gondii* infection, and the acute/chronic stage of the infection. From 2008 to 2018, varying seroprevalence levels have been reported with an increase in proportion.

The method they usually employed is the collection of a blood sample from the study population and examination of their sera for the presence of anti-toxoplasma antibodies. The serological tests frequently used were the ELISA, latex agglutination (LA) test, and IFAT [50,51]. The sensitivities and specificities of commercial tests kits used varied, and their outcome is sometimes not conclusive. Firstly, the measure of the immunoglobulin G (IgG) with seropositive titers at low, higher, or stable determined the chronic stage of the infection, which is also the latent toxoplasmosis [52]. This does not necessarily require monitoring in immunocompetent individuals, but its indication reflects a previous exposure to the infection, and it does not require treatment [34]. In immunosuppressed individuals such as HIV/AIDS patients and pregnant women, IgG seropositive titers at higher level require monitoring and management options to prevent seroconversion, which is a threat to HIV/AIDS patients and the unborn child [53]. Secondly, the immunoglobulin M (IgM) level determines the acute stage of the *T. gondii* infection and may indicate recent exposure to the disease. This is the most serious form which requires treatment and is often regarded as acute toxoplasmosis [52]. In particular, IgM antibodies may be delayed or persist for a long period in the host, which result in either false-positive or false-negative results [35]. Lastly, the IgG avidity measures the exact level of the infection as either acute or chronic infection in combination with IgG or IgM. Low IgG avidity indicates that a recent acute infection cannot be excluded (usually less than 4 months), but it may persist at low level after 4 months in about 10% of the infected individuals [53,54].

### 4.1. Toxoplasmosis among Pregnant Women in Malaysia and a Link with Other Neighboring Countries

Acute toxoplasmosis in pregnant women may likely lead to abortion, stillbirth, hydrocephaly, and several deformities leading to fetus malformation [39]. In Malaysia alone, the seroprevalence of *T. gondii* infection in pregnant women has been reported [35]. The study indicated IgG seroprevalence of 35.2% among pregnant women, which is a sign of past exposure to *T. gondii*. This figure was high compared to the two studies carried out in Thailand, which is a neighboring country (Figure 2) where 25.0% and 28.3% seroprevalence were recorded [55,56]. Such a difference in *T. gondii* seroprevalence could have possibly resulted from the differences in the sample size, condition of the women tested, and the choice of the diagnostic test used between the two countries. In a much broader way, transmission patterns may likely vary, and people are subjected to a different level of exposure to the *T. gondii* infection. Similarly, a study conducted between Malaysian and Burmese (Myanmar) pregnant women, have found a seroprevalence of 42.5% and 30.7%, respectively [36]. Although, all these studies have identified contacts with cats, drinking untreated water, and advanced age as a risk factor for contracting *T. gondii* infection, still much needs to be done to unravel the reason for the high seroprevalence among Malaysians.

Furthermore, a study on *T. gondii* infection among pregnant women located at an unhygienic refugee camp along the Thailand–Myanmar border found a seroprevalence of 31.8% [57]. This situation of lower prevalence even at an unhygienic refugee camp may likely represent the true picture of higher seroprevalence of *T. gondii* antibodies among pregnant women in Malaysia compared to the other three Southeast Asian countries. The main route of acquiring *T. gondii* infection is by ingesting oocysts shed together with cat feces to the outside environment, which can virtually contaminate soil, water, and vegetables [58]. Malaysians have the habit of keeping domestic cats (definitive host) as a pet, and a large number of stray cats roam the street, which may contribute to the spread of *T. gondii* infection. Seroprevalence of *T. gondii* IgG antibodies in Malaysian pregnant women is quite high compared to that in their neighboring countries (Table 2). The findings from Malay, Thai, and Filipino pregnant women on their knowledge and practices toward toxoplasmosis revealed a lack of knowledge about the route of *T. gondii* transmission [59]. Surprisingly, the results highlighted a better understanding of the *T. gondii* infection among Malay and Thai pregnant women compared to their Filipino counterparts. Sociodemographic factors may likely play a role for *T. gondii* transmission among the Filipino pregnant women, while abortion in Thai pregnant women is linked to changing the cat litter box (Table 2) [59]. In general, a better understanding of the route of *T. gondii* infection, awareness on the risk factors, an early diagnosis may contribute to saving the life of the unborn child.

### 4.2. Toxoplasmosis among Human Patients Presenting with Various Disorders in Malaysia

Within the period under review, 3 studies which involved seroprevalence of *T. gondii* among patients presenting with different ailments in a hospital were documented (Table 3). The first study involved group of confirmed renal patients tested for the *T. gondii* antibodies and their seroprevalence was found to be 31.6% [5]. The second study, focused on seroprevalence status of patients with other disorders: 39.5% [56], while the last study tested *T. gondii* seroprevalence among human patients presenting with clinical signs suspected of toxoplasmosis. After they were screened for toxoplasmosis, the result showed that 44.2% [37] had *T. gondii* antibodies. These findings were still within the range reported from healthy individuals of the indigenous community/Orang Asli: 37.0% [39] and people having close contact with animals such as veterinary technicians: 33.3% [46]. In comparison, these groups of human patients who presented with disorders may likely be prone to the *T. gondii* infection, and therefore, chances of opportunistic infection exhibited by the parasite could have played a significant role in the development of pulmonary toxoplasmosis or other clinical conditions [22]. Intriguingly, the findings from Nissapatorn et al. [56] and those from Ngui et al. [39] reported those who were 12 years old and higher as being susceptible to and at risk of acquiring the *T. gondii* infection. Conversely, the study by Nimir et al. [5] and that from Mohamed and Hajissa [37] reported high seroprevalence in younger children and in those below one month of age, which may likely represents a previous exposure to *T. gondii* infection by their mothers as IgG can cross the maternal–fetal barrier [53]. 

### 4.3. Toxoplasmosis among Migrant Workers in Malaysia

From 2008 to 2018, the population of migrants/foreign workers has increased in countries that are relatively peaceful because of conflict in other regions (Table 4). In Malaysia, since the 1980s, the number of migrant workers has increased, and the chances are that diseases not known in the country may have probably been imported [42]. There are at least two studies that have been conducted in Malaysia among migrant workers to ascertain the impact and health status of this group of people. The first report in 2008 indicated a seroprevalence of *T. gondii* at 34.1% and 44.9% among migrant workers and the indigenous population, respectively [42]. The infection in the later was significantly higher at *p* = 0.0009. The second report in 2017 placed the seroprevalence at 57.4% among migrant workers [41]. This was extremely high and could have nearly doubled the figure in the first report. The difference could be because the first report focused only on a single unit of workers at the plantation camp, while the other comprised migrants from different places of work. In addition, results could be biased due to poor sample size and the choice of diagnostic test. However, the second report did not take into account the control group from the local population to allow for meaningful comparison. Nevertheless, findings from these studies have shown a tremendous increase in the seroprevalence level. Further, both studies indicated a high level of seropositivity among Nepalese workers (46.2%) and also from illegal Indonesian workers (54.4%). Findings of *T. gondii* IgG antibodies may indicate previous infection from home countries of the migrant workers, but those with IgM antibodies represent recently acquired infections and likely got it at their place of work [53]. More effort is needed to identify the possible source for *T. gondii* infection to reduce transmission and spread of the disease.

### 4.4. Toxoplasmosis among Healthy Community Members in Malaysia

The highest seroprevalence rate of *T. gondii* so far in Malaysia was recorded among healthy individuals. The study among 345 people of Pangkor Island (Table 5) revealed a seroprevalence of 59.7% [40]. Furthermore, as noted in their published paper, this is the first report that documented higher IgM compared to IgG, indicating a recent exposure with active transmission in Pangkor Island. Acute toxoplasmosis is a serious health condition if untreated, and members of the community, particularly fetuses of pregnant women and immunosuppressed individuals, are likely to have serious complications [7]. Nevertheless, the study found *T. gondii* as the highest protozoan parasite identified from blood in the community. In addition, drinking untreated water and consumption of undercooked meat could be the possible cause of *T. gondii* infection. This is in agreement with previous findings [21], which documented a higher *T. gondii* seroprevalence among Malays compared to other ethnic groups. The standard of living conditions in these communities, as well as personal hygiene, need to be improved to curtail the high rate of *T. gondii* infection [40].

### 4.5. Toxoplasmosis among Schizophrenics in Malaysia

*T. gondii* prefers to reside in the brain of its intermediate host and, consequently, it may lead to some psychiatric disorder [60]. Rat is among the intermediate hosts of *T. gondii* and research has shown the ability of the parasite to manipulate the behavior of its host [61,62]. The parasite also has the ability to reduce the gray matter density of schizophrenic individual patients [63]. Schizophrenia is a psychiatric disease with unknown etiology, and some researchers believe *T. gondii* may likely play an important role in its pathophysiology [64]. In Malaysia, few studies (Table 6) were conducted on the seroprevalence of *T. gondii* antibodies among schizophrenic patients [43,44]. They found almost similar results of 51.0% and 51.5% in Hospital Kajang and Hospital Sungai Buloh respectively, in Selangor, Malaysia. The PCR results from the blood samples collected among the schizophrenic patients compared to control at Hospital Sungai Buloh were significantly different. However, there is a need to be cautious, because the high *T. gondii* prevalence could reflect possible PCR contamination [29]. Nevertheless, both studies found a strong association between *T. gondii* and schizophrenia and further concluded that it might likely be the etiological agent. Much effort is highly required to elucidate further the mechanism of action of this parasite, which will unravel the mystery behind such association. The schizophrenic patient’s blood group and the type of parasite strain may likely shed more light on this behavior deficit. Notably, confirmatory tests such as dye test or IFAT were not performed [53].

### 4.6. HIV/AIDS Patients and Rare Toxoplasmosis in Malaysia—A Case Report

The clinical features of toxoplasmosis in immunocompromised patients, such as HIV patients are not specific and may likely mimic other signs that can lead to an erroneous outcome. The usual signs are fever with neurological imbalance [45]. The problem is more pronounced if it occurs in immunocompromised patients as an opportunistic infection. The case reports on 31- and 49-year-old HIV positive patients in Malaysia are of serious concern [45,65]. The ELISA IgG level was found to be very high and histological, or magnetic resonance imaging (MRI) scan revealed brain abscess/lesions that are suggestive of cutaneous and cerebral toxoplasmosis. There was no detailed report on clinical follow-up and confirmatory test by PCR was not performed. This is a rare situation in Malaysia, and such cases may likely go undetected. The reliable way of monitoring *T. gondii* infection is to incorporate the anti-toxoplasma IgG serological test coupled with histological stain or MRI scan of brain section to monitor HIV/AIDs patients [7].

## 5. Animal Seroprevalence of Toxoplasmosis and a Risk of Zoonotic Diseases in Malaysia

Animals serve an important position in the food chain, which provides a source of nutrients to other animals and humans. Some animals may carry the bradyzoite cyst within tissues of their body and the parasite can subsequently be transmitted to other respective hosts through consumption of raw or undercooked portion of this infected tissues [3]. In this context, a certain group of people such as hunters, butchers, and consumers may likely become infected through consumption of domestic or wild meat which increases the chances of zoonotic infection via foodborne pathogens [25]. It is also notable that the oocyst infective stage is excreted only by wild and domestic cats that cause *T. gondii* infection when consumed in contaminated food, water, or vegetables [17].

### 5.1. Domestic Animals

In Malaysia, having analyzed the available reports in the last decade (2008–2018), the seroprevalence rate of *T. gondii* as zoonotic infection among domestic animals is significantly low and decreasing in prevalence rate. The prevalence level was initially recorded at pigs: 0%, dogs: 9.6%, cats: 14.5%, goats: 35.5%, and cattle: 6.3% [48] compared to a recent and different group of cattle: 2.6% [47]. This decrease in prevalence level may likely be true, but there is a need to be cautious since only cattle were compared with no other subsequent available published reports on pigs, dogs, cats, and goats. Further, it was documented that goats and sheep were susceptible to *T. gondii* infection, which can lead to the abortion of fetuses, while cattle, horses, and buffalo were resistant to the infection [17]. Therefore, subsequent study needs to be carried out on pigs, sheep, and goats to check their status on the chances of zoonotic infection. 

### 5.2. Wild Animals

The threat of zoonotic infection from wild animal meat has been reported [49]. The results suggest that *Sarcocystis*, *Toxoplasma*, and *Trichinella* species are frequently found in wildlife meat compared to the meat sourced from domestic animals. Since their lifecycles involve multiple wildlife hosts, the wild meat trade may increase the risk of zoonotic transmission, via foodborne or fecal–oral routes [25]. In spite of this growing threat from wild animal meat, its consumption is still in practice in some remote areas [30]. On the other hand, the study found a PCR prevalence of 4.3% from exotic meat, mostly from monkey and squirrel. Therefore, research in these wild animals should be of consideration, since the former serves as the main source of nutrients as well as pets, while the latter is mainly for consumption [66].

### 5.3. Rodents and Other Species

Research on the rodent intermediate host was scarce, and only a single report was documented [49]. They collected the blood of 526 rodents, tested for *T. gondii* antibodies using IFAT, and found 5.9% were infected. In addition, commensal rats, *Rattus exulans,* were more infected compared to squirrels. Similarly, Nimir [67] captured 100 rats and also collected water samples from hawkers at a wet market. Tissue cysts suggestive of *T. gondii* were found in the brain of three rats. Unfortunately, they could not recover *T. gondii* oocysts from all the water samples. However, in these studies, no confirmation of positive samples was done by either PCR or bioassay in mice or cat. 

### 5.4. Wild and Domestic Cat

Both wild and domestic cats are the only definitive host and source of *T. gondii* oocysts which can spread the infection to all mammal and bird intermediate host [17]. Malaysian and, in particular, Malay ethnic group are known to keep domestic cats as pet, while camera-trapping has shown a wide distribution of wild cats in the forest, which are predominantly clouded leopards (*Neofelis diardi*) [68]. Unfortunately, there is no single report on *T. gondii* on either wild or domestic cats during 2008–2018 from Malaysia. 

## 6. Innovative Diagnostic Approach and Vaccine Development for *T. gondii* in Malaysia

A vaccine against *T. gondii* presents a difficult task with its complex life cycle, stage-specific variation, different strains, and perhaps virulence effect. The acute infection is always the target because the parasite is in the tachyzoite form, which is the active multiplication stage and is found in the blood, which reflects recent exposure [54]. This is always fatal in immunosuppressed individuals particularly fetuses of pregnant women in their first trimester because the conversion of tachyzoite to the bradyzoite stage will be delayed [52]. Furthermore, the tachyzoite can move via the placenta to the fetus and causes severe damage such as abortion, stillbirth, hydrocephalus, etc. Chronic stage or latent toxoplasmosis involves the bradyzoite, which is a slow or dormant stage found in the tissues of the liver, heart, and mainly the brain of the infected host indicating past exposure [69]. Most of the bradyzoites may likely reconvert back to tachyzoites in immunosuppressed individuals such as HIV/AIDS patients causing pulmonary toxoplasmosis or toxoplasma encephalitis [22,60]. However, the development of pulmonary toxoplasmosis and other clinical conditions in immunocompetent individual depends on the *T. gondii* strain causing the infection [6]. Unfortunately, most of the currently available test kits in the market cannot accurately differentiate between the anti-toxoplasma antibodies of IgM or IgG, leading to the erroneous conclusion of either false-positive or false-negative results [7]. While the search for novel drugs that are safe to treat both tachyzoite and bradyzoite continues, accurate diagnosis remains elusive. 

Recently, the ability to diagnose infection falls largely on the potential of the technique to be able to diagnose the infection properly. In Malaysia, within the period under review, several recombinant proteins were produced and tested for their potential as diagnostic antigens of *T. gondii* infections. These antigens included the excretory–secretory antigens (ESA), surface antigens (SAG1, SAG2), rophtry antigens (ROP1, ROP8), and dense granule proteins (GRA2, GRA5, GRA7). The results obtained from works on the improved methods for diagnosis varied among the laboratories [27,52,54,70,71,72,73,74,75,76,77,78]. They reported different levels of sensitivities and specificities of these markers. This may be due to variation within the antigens, the difference in *T. gondii* strain used, limited samples evaluated, the expressing vector, and the nature of the serum sample. Nevertheless, a combination of multiple antigens rather than single antigen always produced promising results [20]. In this context, the diagnostic markers employed for *T. gondii* need to be of high sensitivity and specificity to be able to differentiate between acute and chronic infection. In the future, efforts should be made toward the development of simple, sensitive, inexpensive, rapid lateral flows, which can be used in remote areas that have limited access to sophisticated equipment. Lastly, protocols need to be standardized to allow for repeatability and reproducibility among laboratories.

Several approaches have been considered in the last decade for the development of the human *T. gondii* vaccine. These range from DNA vaccines and recombinant proteins vaccine to inactivated vaccines, which can elicit a long-term immune response [72]. The excellent review by Lim and Othman [79] captured the essence of conducting such research. These studies proposed a blueprint on the development of a human vaccine against *T. gondii*. They highlighted that not only survival rate should be measured, but reduction/elimination of the number of cysts also has to be considered. Search from the available literature revealed sparse research in these field [20,72,77,80,81,82,83]. Despite some hiccups, some of the results showed remarkable achievements, which not only increased the survival rate but also elicited cell-mediated immunity that triggered humoral immunity. Although sterile immunity was not fully achieved, effort should be made toward a multi-epitope vaccine targeting several regions of the proteins that can hinder tachyzoite multiplication and block its conversion to bradyzoite. 

The results obtained in the different research works reporting on the pathogenesis, immunogenicity, and treatment of *T. gondii* are worthy to be contextualized. The articles relative to Malaysia indicated some suitable proteins markers that can serve either for treatment or as good candidates for vaccine development [70,71,72,77,83,84]. Additionally, the fact that some of the drugs for the treatment of *T. gondii* are teratogenic and still no cure for the bradyzoite tissue cysts must be taken into consideration. Notwithstanding, the negative impact *T. gondii* infection may have on HIV/AIDS patients and pregnant women, coupled with the risk factors, make it necessary to continue to search for a collective alternative approach precisely. In general, a correct diagnosis and proper treatment have been identified as the key factors to mitigate the spread of infection. Nonetheless, those measures may not be sufficient to eliminate or eradicate the infection without looking at the risk associated with the infection. After the screening of a community in Pangkor Island in Peninsular Malaysia, it was observed that females were mostly infected (64.7%) compared to males (52.8%) [40]. In addition, Malays were highly infected (66.0%) compared to Indian (40.0%) and Chinese (28.3%) ethnic groups. Moreover, the findings of higher IgM antibodies than IgG ones signal active transmission going on in that community. Therefore, in any case, research looking after vulnerable communities, pregnant women, and HIV/AIDS patients should take *T. gondii* into serious consideration in order to achieve early diagnosis and prevent the development of chronic clinical conditions [85,86].

## 7. Genetic Diversity and Population Structure of *T. gondii* in Malaysia 

The geographic location where animals are found varies greatly in the world, which also shapes the distribution and genetic characterization of *T. gondii* [87]. The population structure of *T. gondii* in Europe and North America is unique and has been found to be from three clonal lineages [88,89,90]. Mixed genotypes of exotic or atypical strains in addition to the three clonal lineages were reported in South America [10,91,92]. The African and Asian continents were the least explored. In particular, pockets of research in Asian countries have documented patterns similar to the ones in South America [93,94,95,96,97]. Only two studies were carried out in Malaysia to genotype *T. gondii* strains (Table 7). One of the reports highlighted the presence of the type I strain in wild boar meat [98]. These findings are similar to those obtained from bats in Myanmar [87]. The study was conducted at the Myanmar–China border, and the former also shares a border with Malaysia. These findings may likely present a bigger picture than expected as it is well documented in a review of genotypes in Asia [99]. Bat and wild boar meats are consumed by some people, as a delicacy. The type II strain has been shown to be less virulent in a mice murine model and is the predominant strain isolated from immunocompromised patients [22]. Several toxoplasmosis infections such as ocular, congenital, and cerebral toxoplasmosis have been directly correlated with type I strain [100,101,102,103]. This trend can facilitate the transmission of toxoplasmosis as a zoonotic infection, which is highly critical in immunosuppressed individuals. There is an urgent need to further explore the population structure of this organism that is circulating in this region. 

## 8. Conclusions

Findings in the present review on toxoplasmosis research from 2008 to 2018 in Malaysia indicated limited data on animal seroprevalence. The seroprevalence among pregnant women in Malaysia is on the increase, but little is known on clinical congenital toxoplasmosis. Thus, no information exists on the mode of *T. gondii* transmission from the domestic cat definitive host despite their huge number as either pets or free-roaming cats. In addition, little is known on the seroprevalence of toxoplasmosis in livestock, particularly chicken destined for human consumption. Furthermore, genetic characterization to determine the *T. gondii* strain circulating in Malaysia from HIV patient, pregnant women, livestock, and domestic cat is lacking. Reports on toxoplasmosis among migrant workers and rural communities in Malaysia is alarming. This calls for the inclusion of toxoplasmosis test at the point of entry into Malaysia to screen acute infection and also the need to educate the local community on the risk associated with *T. gondii* transmission. This present review on toxoplasmosis is beneficial to researchers, health workers, veterinary attendants, and policymakers. Therefore, urgent attention is required to educate and enlighten people about the risk factors associated with toxoplasmosis infection in humans and animals. This can be achieved through health education and campaigns in local newspapers, televisions, radios, and more recently using social media outlets.

## Figures and Tables

**Table 1:** Serological methods used for the detection of *T. gondii* antibodies in humans and animals in Malaysia.

Host	Specimen	Sample Size	Test	Manufacturer	Interpretation of Results	Reference
Pregnant women	Blood	281	ELISA	BioRad, USA	IgG < 6 IU/mL negative, IgG < 6 IU/mL and 9 IU/mL equivocal, IgG > 9 IU/mL positive	[35]
Pregnant women	Blood	215	ELISA	IgG & IgM-NovaLisa Dietzenbach, Germany	IgG, IgM and Avidity High avidity past infection (4–5 months). Low avidity, recently acquired (4–5 month)	[36]
Patients with disorder	Blood	102	ELISA	Bio-Rad, USA	IgG < 0.80 negative, IgG ˃1.0 positive Avidity; <20% acute	[37]
Patients with disorder	Blood	129	IFAT	na	IgG > 51 IU/mL positive IgM > 51 IU/mL positive	[5]
Renal patient	Blood	247	ELISA	IgG-Trinity Biotech, New York, NY and IgM-Trinity Biotech, New York, NY)	IgG > 51 IU/mL positive IgM > 51 IU/mL positive Avidity; <40% latent	[38]
Healthy community members	Blood	495	ELISA	Trinity Biotech, New York, NY	IgG > 51 IU/mL positive IgM > 51 IU/mL positive Avidity; <40% latent	[39]
Healthy community members	Blood and stool	345	ELISA	Trinity Biotech, USA	IgG and IgM	[40]
Migrant workers	Blood Venous/plain	484		Trinity Biotech Captia^TM^, New York, USA	IgG ≥ 1.23, positive IgM ≥ 1.23, positive Avidity’ ˃40% latent and ≤40% acute	[41]
Migrant workers	Blood	Migrants, 501 Control 198	IFAT and ELISA (IgM)	na	1:64 significant titers. Positive samples diluted two-fold, end point values. IgM dilution 1:100	[42]
Schizophrenic	Blood	Schizophrenic (88) Control (88)	ELISA	RADIM, Italy	na	[43]
Schizophrenic	Blood	cases (101) control (55)	ELISA and qPCR	IBL company, Hamburg Germany	IgG and IgM positive more than IU/mL and 11 IU/mL	[44]
HIV patient	Blood	1	ELISA	na	IgG > 51 IU/mL as positive	[45]
Veterinary health professionals & students	Blood	312	ELISA	IgG-NovaLisa, Dietzenbach, Germany	ELISA-I, II and III Avidity >40%, chronic, <40% acute. Avidity of <15% (low avidity), acute primary infection Avidity between 15% and 30% (borderline)	[46]
Cattle	Blood	116	IFAT	na	1:20	[47]
Domestic animals	Blood	Cat (55), dog (135), goats (200) cattle (126) Pigs (100)	IFAT	na	na	[48]
Rats	Blood	526	IFAT	na	IgG ≥ 1.64 IgM ≥ 1.4	[49]

ELISA: enzyme-linked immunosorbent assay, IFAT: indirect fluorescent antibody test, qPCR: quantitative real-time, polymerase chain reaction, IgG: immunoglobulin G, IgM: immunoglobulin M, DNA: deoxyribonucleic acid, na: not available.

**Table 2:** Serological prevalence of toxoplasmosis among pregnant women in Malaysia and a link with other countries.

Country	Year	G	Design	Subject	Sample Size	S	Test	Findings	Reference
Malaysia	2014	Female	Cross-sectional	Pregnant women	281	Sera	ELISA	IgG: 35.2%, IgM/IgG: 1.8%, IgG avidity: high and low. The Toxoplasma antibody was confirmed through IgG avidity index, and it was found to be accurate.	[35]
Malaysia and Myanmar	2014	Female	Cross-sectional	Pregnant women	219 Malaysia, 215 Myanmar	Sera	ELISA	Malaysia, IgG: 39.7%, IgG and IgM: 2.7%. Myanmar, IgG: 30.2%, IgG and IgM: 0.5%. Malaysian pregnant women were more prone to *T. gondii* infection in the age group 30 years and above. Lack of awareness was also associated with the infection.	[36]
Thailand and Myanmar border	2017	Both	Cross-sectional	Pregnant women/ Refugee	200	Sera	ELISA	IgG: 31.7%, IgM: 1.5%, IgG avidity: high. The infection is more prevalent in people aged 35 years and above. Similarly, being Muslim was associated with a higher risk of infection.	[57]
Thailand	2011	Female	Cross-sectional	Pregnant women	640	Sera	ELISA	IgG: 21.6%, IgM and IgG: 6.7%, IgG avidity: high. Contact with cats and untreated water are the confirmed risk factors.	[56]
Thailand	2014	Female	Cross-sectional	Pregnant women	760	Sera	ELISA	IgG: 22.0%, IgM: 3.0% IgG avidity: high. Pregnant women 26 years and above working as laborers and untreated water were identified as risk factors.	[55]

G: gender; S: sample collected; IFAT: indirect immunofluorescence antibody test; ELISA: enzyme-linked immunosorbent assay; LAT: latex agglutination test; IgG: immunoglobulin G; IgM: immunoglobulin M.

**Table 3:** Serological prevalence of toxoplasmosis among patients presenting with various disorders in Malaysia.

Country	Year	G	Design	Subject	Sample Size	S	Test	Findings	Reference
Malaysia	2010	Both	Cross-sectional	Patients with disorders	129	Sera	ELISA	IgM: 0.8%, IgG: 38.8%, IgM and IgG: 2.3%. Highest seroprevalence in the age group 20–41 years old. Malay has the highest IgG positivity (32.0%) and the least is among Indians (1.0%). The younger population are at risk of infection.	[5]
Malaysia	2011	Both	Cases	Renal patients	247	Sera	ELISA	IgG: 31.6%. High prevalence of latent toxoplasmosis in renal patients with older people at high risk.	[56]
Malaysia	2016	Both	Cross-sectional	Patients with disorders	102 cases	Sera	ELISA	IgG: 44.1%, IgM: 1.0%. The highest was recorded among patients screened for congenital toxoplasmosis (41.7%) and in children less than one-month-old (37.8%).	[37]

G: gender; S: sample collected; ELISA: enzyme-linked immunosorbent assay; IgG: immunoglobulin G; IgM: immunoglobulin M.

**Table 4:** Serological prevalence of toxoplasmosis among migrant workers in Malaysia.

Country	Year	G	Design	Subject	Sample Size	S	Test	Finding	Reference
Malaysia	2008	Male	Cross-sectional	Foreigners/Migrant workers	501 Migrants, 198 local Malaysians, 90 Police and Immigration	Sera	IFAT	Migrants—IgG: 34.1%, IgM: 5.2%; Locals—IgG: 44.9%, IgM: 8.6%. All nationalities were seropositive with *T. gondii* antibodies. Seroprevalence among illegal Indonesian workers was the highest, 54.4%, compared to that among Nepalese workers, 46.2%. Infection not imported, those infected got it at the plantation camp.	[42]
Malaysia	2017	Both	Cross-sectional	Migrant workers	484	Sera	ELISA	IgG: 52.9%, IgM: 0.8%, IgG and IgM: 3.8%, IgG avidity: high. The most common factor associated with the prevalence of the infection is age class. Workers older than 45 years and above were found to be more prone to the infection. Second is the migrant countries of origin, which was also identified as a risk factor.	[41]

G: gender; S: sample collected; ELISA: enzyme-linked immunosorbent assay; IgG: immunoglobulin G; IgM: immunoglobulin M.

**Table 5:** Serological prevalence of toxoplasmosis among healthy community members in Malaysia.

Country	Year	G	Design	Subject	Sample Size	S	Test	Findings	Reference
Malaysia	2011	Both	Cross-sectional	Orang Asli/Indigenous	495	Sera	ELISA	IgG: 31.0%, IgM: 1.8%, IgG and IgM: 4.2%, IgG avidity: low. Seroprevalence higher among 12 years and above. Close contact with cats and untreated water as risk factors.	[39]
Malaysia	2014	Both	Cross-sectional	Pangkor Island	345 individuals	Stool and Sera	Micros and ELISA	*Trichuris trichiura*: 5.3%. IgG and IgM: 59.7%. Seroprevalence was 59.7% with the infection being higher in children, in females, and also in Malays compared to Indians and Chinese.	[40]
Malaysia	2015	Both	Cross-sectional	Veterinary personnel and pet owners	312 people: Veterinarian Technicians Students	Sera	ELISA	IgG: 18.3%, IgM: 1.0%, IgG and IgM: 0.7%, IgG avidity: low, but no clinical symptoms. Veterinarians: 18.4%, technicians: 33.3%, students: 14.9%, and pet owners: 31.4%. Technicians had the highest risk and vulnerability to the infection. Working duration, age group (above 30 years), and gardening were risk factors. Indians were the highest infected 29.0%.	[46]

G: gender; S: sample collected; ELISA: enzyme-linked immunosorbent assay; IgG: immunoglobulin G; IgM: immunoglobulin M; Micros, microscopy.

**Table 6:** Serological prevalence of toxoplasmosis among schizophrenics in Malaysia.

Country	Year	G	Design	Subject	Sample Size	S	Test	Findings	Reference
Malaysia	2015	Both	Case control	Schizophrenic	101 Schizophrenic patients 55 control	Sera	ELISA and qPCR	Schizophrenic—IgG: 51.5%, IgM: 3.9% DNA: 32.1%; Control—IgG: 18.2%, IgM: 0%, DNA: 3.6%. The study confirmed strong association between *T. gondii* and schizophrenia.	[44]
Malaysia	2013	Both	Case-control	Hospital-based	88 Schizophrenic 88 Control	Sera	ELISA	Schizophrenic—IgG: 51.0%, IgM: 1.1%; Control—IgG: 30.7%, IgM: 1.1%. Beef and pork consumption, and risky cats were significantly associated with the infection. There is an association between *T. gondii* and schizophrenia.	[43]

G: gender; S: sample collected; ELISA: enzyme-linked immunosorbent assay; qPCR: quantitative real-time polymerase chain reaction; IgG: immunoglobulin G; IgM: immunoglobulin M, DNA: deoxyribonucleic acid.

**Table 7:** Shows reported genotypes of *T. gondii* by polymerase chain reaction-restriction fragment length polymorphism PCR-RFLP in Malaysia other countries in Southeast Asia.

Country	Genotype	Host	Sample Used for Genotyping	Number of *T. gondii* Isolates	Reference
Indonesia	Non clonal	Chicken	Bioassay in mice	1	[104]
Vietnam	Non clonal	Chicken	Bioassay in mice	1	[104,105]
Myanmar	type I	Bats	Direct from organs	19	[87]
Malaysia	type I and II	Ducks	Bioassay in mice	4	[106]
Malaysia	type I	Wild Boars	Bioassay in mice	11	[98]
Thailand	type I, III, II or III and recombinant	domestic cat	Direct from feces	13	[94]

## Abbreviations

The following abbreviations are used in the manuscript:

AIDS	Acquired immune deficiency syndrome
ELISA	Enzyme-linked immunosorbent assay
ESA	Excretory–secretory antigens
GRA	Dense granule proteins
H&E	Hematoxylin and eosin
HIV	Human immunodeficiency virus
IFAT	Indirect fluorescence antibody test
IgG	Immunoglobulin G
IgM	Immunoglobulin M
LAT	Latex agglutination test
LMAP	Loop-mediated amplification
MAT	Modified agglutination test
MRI	Magnetic resonance imaging
PCR	Polymerase chain reaction
PCR-RFLP	Polymerase chain reaction—restriction fragment length polymorphism
ROP	Rophtry antigens
SAG	Surface antigens
T. gondii	*Toxoplasma gondii*
qPCR	Quantitative-PCR
